# Effects of a standardized community health worker intervention on hospitalization among disadvantaged patients with multiple chronic conditions: A pooled analysis of three clinical trials

**DOI:** 10.1111/1475-6773.13321

**Published:** 2020-07-08

**Authors:** Aditi Vasan, John W. Morgan, Nandita Mitra, Chang Xu, Judith A. Long, David A. Asch, Shreya Kangovi

**Affiliations:** ^1^ National Clinician Scholars Program University of Pennsylvania Perelman School of Medicine Philadelphia Pennsylvania; ^2^ PolicyLab and Department of Pediatrics Children's Hospital of Philadelphia Philadelphia Pennsylvania; ^3^ Leonard Davis Institute of Health Economics University of Pennsylvania Philadelphia Pennsylvania; ^4^ Division of General Internal Medicine University of Pennsylvania Perelman School of Medicine Philadelphia Pennsylvania; ^5^ Department of Biostatistics, Epidemiology, and Informatics University of Pennsylvania Perelman School of Medicine Philadelphia Pennsylvania; ^6^ Corporal Michael J Crescenz VA Medical Center Center for Health Equity Research and Promotion Philadelphia Pennsylvania; ^7^ Penn Center for Community Health Workers University of Pennsylvania Perelman School of Medicine Philadelphia Pennsylvania

**Keywords:** access/demand/utilization of services, determinants of health/population health/socioeconomic causes of health, internal medicine, social determinants of health

## Abstract

**Objective:**

To analyze the effects of a standardized community health worker (CHW) intervention on hospitalization.

**Data Sources/Study Setting:**

Pooled data from three randomized clinical trials (n = 1340) conducted between 2011 and 2016.

**Study Design:**

The trials in this pooled analysis were conducted across diseases and settings, with a common study design, intervention, and outcome measures. Participants were patients living in high‐poverty regions of Philadelphia and were predominantly Medicaid insured. They were randomly assigned to receive usual care versus IMPaCT, an intervention in which CHWs provide tailored social support, health behavior coaching, connection with resources, and health system navigation. Trial one (n = 446) tested two weeks of IMPaCT among hospitalized general medical patients. Trial two (n = 302) tested six months of IMPaCT among outpatients at two academic primary care clinics. Trial three (n = 592) tested six months of IMPaCT among outpatients at academic, Veterans Affairs (VA), and Federally Qualified Health Center primary care practices.

**Data Collection/Extraction Methods:**

The primary outcome for this study was all‐cause hospitalization, as measured by total number of hospital days per patient. Hospitalization data were collected from statewide or VA databases at 30 days postenrollment in Trial 1, twelve months postenrollment in Trial 2, and nine months postenrollment in Trial 3.

**Principal Findings:**

Over 9398 observed patient months, the total number of hospital days per patient in the intervention group was 66 percent of the total in the control group (849 days for 674 intervention patients vs 1258 days for 660 control patients, incidence rate ratio (IRR) 0.66, *P* < .0001). This reduction was driven by fewer hospitalizations per patient (0.27 vs 0.34, *P* < .0001) and shorter mean length of stay (4.72 vs 5.57 days, *P* = .03). The intervention also decreased rates of hospitalization outside patients' primary health system (18.8 percent vs 34.8 percent, *P* = .0023).

**Conclusions:**

Data from three randomized clinical trials across multiple settings show that a standardized CHW intervention reduced total hospital days and hospitalizations outside the primary health system. This is the largest analysis of randomized trials to demonstrate reductions in hospitalization with a health system‐based social intervention.

**What this study adds**

- Policymakers are increasingly incentivizing health systems to shift their work upstream and address social determinants of health, but it remains unclear how to design and implement programs that both target social needs and reduce hospitalizations.
- In this pooled analysis of three randomized clinical trials across multiple settings, we found that a standardized community health worker intervention reduced total hospital days and hospitalizations outside the primary health system among a population of socioeconomically disadvantaged patients with multiple chronic conditions.
- Our findings demonstrate that a holistic, tailored, longitudinal, and theory‐based intervention can be effective in reducing hospitalizations and decreasing fragmentation of acute care, both desirable but elusive goals for health system‐based social interventions.

## 1 INTRODUCTION

In the United States, hospitalizations account for one third of all healthcare expenditures, amounting to more than 1.1 trillion dollars annually.^1^ Hospital‐based care is often both costly and inefficient, as it is focused on managing the complications of chronic conditions rather than addressing their underlying causes. Low‐income and publicly insured patients are more likely to be hospitalized^2^ and to experience fragmentation of acute care, including readmissions to two or more different hospitals.^3^ These hospitalizations across multiple different health systems can lead to redundancies in care and testing, resulting in increased hospital costs, longer lengths of stay, and higher mortality rates.^3^, ^4^, ^5^, ^6^

Policymakers, including the Centers for Medicare and Medicaid Services (CMS), are now incentivizing healthcare organizations to shift their work upstream and focus on the underlying social, economic, and behavioral determinants of health.^7^, ^8^ However, it remains unclear how health systems can most effectively design and implement programs that target patients’ social needs and also reduce hospitalizations. Unfortunately, very few high‐quality studies of health system‐based social interventions have been able to demonstrate both improvements in health outcomes and reductions in healthcare utilization and cost.^9^, ^10^, ^11^, ^12^

One potential strategy is for health systems to recruit, train, and deploy a workforce of community health workers (CHWs), trusted laypeople who often share a socioeconomic background and demographic characteristics with their patients and can therefore provide support consistent with patients’ values and needs. Individualized Management for Patient‐Centered Targets (IMPaCT) is a standardized, exportable CHW model developed using a theory‐based approach informed by qualitative participatory action research with high‐risk patients. IMPaCT has been evaluated in three previous randomized clinical trials conducted across different clinical settings.^13^, ^14^, ^15^ In these prior studies, IMPaCT was shown to improve a variety of outcomes including access to preventive care, self‐reported mental health, patient‐perceived quality of care, and rates of hospitalization, although each individual trial was not powered for this last outcome.

In this study, we used pooled data from these three trials to analyze the effects of this intervention, across diseases and settings, on hospital utilization. As a secondary outcome, we assessed the effect of IMPaCT on fragmentation of acute care, as measured by the proportion of hospitalizations occurring outside a patient's primary health system. As an exploratory outcome, we also examined the effect of IMPaCT on admission acuity as measured by DRG weight, to assess whether the intervention influenced severity of illness.

## 2 METHODS

### 2.1 Original trials

This study is based on pooled patient‐level data from three prior two‐armed, single‐blind, randomized clinical trials of the IMPaCT intervention conducted between April 2011 and March 2016. The designs of these trials, as well as selected clinical outcomes, have been reported previously.^13^, ^14^, ^15^ In all three trials, patients were randomly assigned to receive usual care versus IMPaCT, a standardized intervention in which CHWs, trained and trusted individuals who often share a socioeconomic background with patients, provide tailored coaching, social support, navigation, and advocacy to help low‐income patients achieve their stated health goals.

Trial 1 was conducted at two academically affiliated hospitals in Philadelphia between April 2011 and October 2012.^13^ Hospitalized patients who were publicly insured or uninsured and resided in a high‐poverty region of Philadelphia were eligible to participate. CHWs met patients in the intervention group during their hospital admission and then worked with them for 14‐30 days postdischarge. Trial 2 was conducted at two urban, academically affiliated adult internal medicine primary care practices in Philadelphia between July 2013 and October 2014.^14^ Trial 3 was conducted at three primary care sites in Philadelphia: a Veterans Affairs (VA) primary care practice, a federally qualified health center, and an academic family practice clinic, between January 2015 and March 2016.^15^ In both Trials 2 and 3, patients were eligible to participate if they were uninsured or publicly insured, lived in a high‐poverty region of the city, and had been diagnosed with two or more of the following chronic conditions: hypertension, diabetes, obesity, and tobacco dependence. We incorporated the presence of two or more of chronic conditions as part of our inclusion criteria for these trials both because these conditions are highly prevalent in low‐income populations, and because inadequate management of these conditions frequently leads to hospitalization. In both trials, CHWs initially met with patients during their primary care appointments and then worked with them for the following 6 months.

The IMPaCT intervention consists of three stages: goal‐setting, tailored support, and connection with long‐term support.^16^, ^17^ At the time of enrollment, CHWs used a semi‐structured interview guide to get to know patients and understand the factors impacting their health, including social and behavioral determinants of health such as food insecurity, housing instability, drug and alcohol use, and presence or absence of social support. CHWs and patients then worked together to create individualized, patient‐driven action plans personalized based on each patient's needs and preferences. Action plans were conceptualized based on a broad definition of health as a state of physical, mental, and social well‐being.^18^

CHWs subsequently provided tailored support to help patients execute their action plans. For example, for a depressed single mother who never prioritized self‐care, the CHW arranged for a local beauty shop to provide free services which helped to boost the patient's sense of self‐worth. CHWs worked closely with care teams, communicating through team huddles, telephone calls, or electronic medical record messaging. If patients were hospitalized during the intervention, CHWs also attempted to meet with them during their hospitalizations to provide social support and coordination of care. In the final stage of the intervention, CHWs helped patients identify long‐term supports, such as neighbors, family members, friends, or religious or community organizations that could help support them in meeting their goals after the intervention ended. Importantly, throughout the course of the intervention, CHWs were never tasked with “keeping patients out of the hospital.” Their primary objective was to support patients in meeting their own goals.

Across all three trials, the IMPaCT intervention was highly standardized and structured in its approach to CHW hiring, training, and supervision. IMPaCT CHWs were required to have a high school diploma but were not required to have prior experience working in public health or health care. They were recruited and hired based on behavioral interviews assessing key personality traits, such as empathy.^19^ They underwent a standardized month‐long training process in which they learned about topics such as action planning and motivational interviewing. CHWs were supervised by a manager who provided ongoing support, training, and assistance with clinical integration. Managers typically had a master's degree in social work and were responsible for ensuring intervention fidelity through weekly assessments including audits of documentation and observation of CHWs in the field. Each manager supervised a team of six CHWs deployed across a variety of clinical settings, and each of these teams served approximately 55 patients per CHW for the six‐month primary care‐based intervention utilized in Trials 2 and 3, and 90 patients per CHW for the one‐month posthospitalization intervention utilized in Trial 1. We estimate that the average cost of the CHW intervention across all three trials was approximately $1499 per patient, per year (Table S2). A detailed analysis of expenditures and cost savings associated with the IMPaCT CHW intervention, using data from Trial 2, has been reported elsewhere.^20^

Each of these studies was approved by the appropriate Institutional Review Boards. All participants provided written informed consent.

### 2.2 Outcome measures

The prespecified primary outcome for this pooled analysis was all‐cause hospitalization, as measured by total number of hospital days and number of hospital days per patient. Hospital days per patient were calculated by dividing the total number of hospital days for all patients in the intervention and control groups by the number of patients in each group who were hospitalized. Hospitalization data for patients outside the VA system were collected by linking patient identifiers from each study with the Pennsylvania Health Care Cost Containment Council (PHC4) statewide database for all hospital discharges across Pennsylvania.^21^ This dataset included dates, location, and diagnosis‐related group (DRG) weights for each hospitalization. Hospitalization data for veterans were assessed using the national Veterans Health Administration Corporate Data Warehouse, as VA policies did not permit linking identifiers to hospitalization data outside of the VA firewall. Hospitalization data were collected at 30 days postdischarge in Trial 1, twelve months postenrollment in Trial 2, and nine months postenrollment in Trial 3.

As secondary outcomes, we measured average length of stay, calculated using admission date and discharge date information from the PHC4 database, 30‐day readmission rate, as measured by the proportion of patients with readmission within 30 days of a hospitalization, proportion of patients with multiple hospitalizations, and fragmentation of inpatient care, as measured by the proportion of hospitalizations that occurred outside patients’ primary health system. For Trial 1, we defined patients’ primary health system as the one affiliated with their index hospitalization. For Trials 2 and 3, we defined patients’ primary health system as the one affiliated with their primary care clinic. For this outcome, we excluded Trial 3 patients recruited at the FQHC (as this clinic was not affiliated with a single primary health system), and those recruited at the VA (as our data source did not include information about their hospitalizations outside the VA).

As an exploratory outcome, we examined the effect of the IMPaCT intervention on admission acuity as measured by DRG weight. VA patients were excluded from this analysis, as DRG values were not available for VA hospitalizations.

### 2.3 Statistical analysis

Baseline participant characteristics were compared using chi‐squared tests for categorical variables, two‐sample *t* tests for continuous variables when comparing participants across the two treatment arms, and ANOVA for continuous variables when comparing participants across the three included trials. Incidence rate ratios for hospitalization were generated by dividing the number of hospital days per patient in the intervention arm by the number of hospital days per patient in the control arm. We estimated the acuity of each hospitalization by multiplying its DRG code by standardized case‐mix weights published by the Centers for Medicare and Medicaid Services.^22^ We then averaged these case‐mix weights for the intervention and control groups and divided these by the average case‐mix weight for all Medicaid discharges in our data set. We graphically depicted the timing of hospitalizations during and after the intervention period by plotting the cumulative number of hospitalizations by arm at each day for nine months postenrollment in Trials 2 and 3, for which longitudinal hospitalization data were available.

An intention to treat analysis based on the original randomization was conducted for all outcomes. For each of our primary and secondary outcomes, we used generalized linear mixed‐effects models with the appropriate family and link function specified according to the distributional form of the outcome. For skewed count outcomes, including hospital days per patient, hospitalizations per patient, and mean length of stay, we used mixed‐effects negative binomial regression. For binary outcomes, including proportion of patients with multiple hospitalizations, proportion of hospitalizations with a readmission within 30 days, and proportion of hospitalizations outside the primary health system, we used mixed‐effects logistic regression. For continuous outcomes, including mean DRG weight, we used mixed‐effects linear regression. A likelihood ratio test for the treatment group by trial interaction was used to assess for heterogeneity of treatment effect across the three included trials. All models included an indicator for original trial and site of recruitment and accounted for correlation within trials, using a random intercept model. Additionally, an offset term was used in all models to account for differential durations of follow‐up across all three trials. No baseline variables were imbalanced between treatment arms, and therefore these variables were not included in our regression models. All tests were two‐sided with a significance level set at 0.05. All analyses were conducted in SAS version 9.4.

## 3 RESULTS

### 3.1 Study population

**FIGURE 1:**
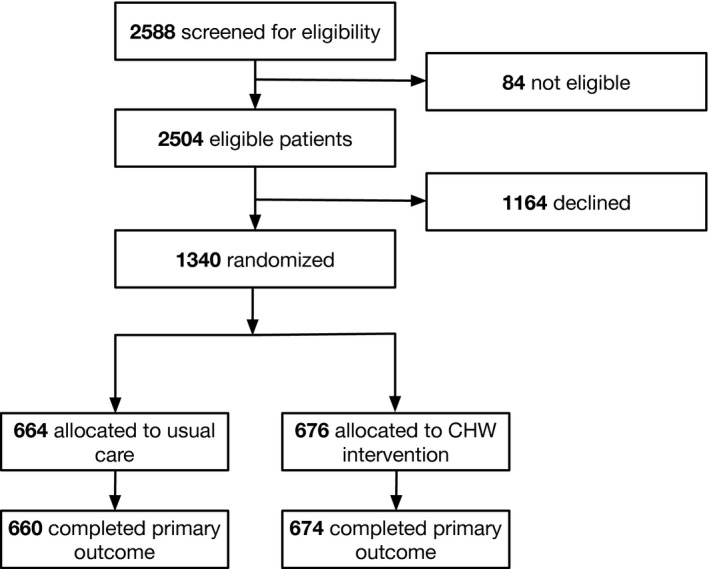
Consort Diagram *Note: *Across all three trials included in this pooled analysis, 2588 individuals were screened for eligibility, 2504 were found to be eligible, and 1340 consented to be part of the intervention. Of these 1340 participants, 664 were allocated to usual care, and 676 to the CHW intervention. Hospitalization data were available for all but 2 of the intervention participants and all but 4 of the control participants.

In total 2588 patients were screened, of whom 84 were ineligible. Of the 2504 eligible patients, 1340 (54 percent) consented and were randomized (446 from Trial 1, 302 from Trial 2, and 592 from Trial 3). As compared to nonparticipants, participants were younger (mean age 50.8 years vs 56.7 years; *P* < .001) and more likely to be female (863 [64.4 percent] vs 628 [54.1 percent]; *P* < .001). Of the patients who consented to participate, 676 were randomized to the IMPaCT intervention and 664 received usual care (Figure 1). Baseline characteristics were similar between intervention and control groups (Table S1). This was a middle‐aged (51.3 vs 50.3 years, *P* = .08), predominantly black (93.8 percent vs 94.4 percent, *P* = .63), and predominantly publicly insured (70.8 percent vs 73.5 percent, *P* = .27) population, most of whom had a household income of less than $15 000 (72.7 percent vs 72.4 percent, *P* = .84). More than half of patients in both groups had at least one hospitalization in the prior 12 months (53.4 percent vs 54.0 percent, *P* = .29) with a similar average number of hospitalizations per patient in the year prior between groups (1.4 vs 1.9, *P* = .72).

Table 1 shows variation in the demographic characteristics of study participants by trial. Characteristics with notable variation across trials 1, 2, and 3, respectively, include proportion of uninsured participants (69.3 percent vs 17.9 percent vs 2.2 percent, *P* < .0001) and proportion with one or more hospitalizations in the previous 12 months (90.5 percent vs 35.0 percent vs 35.8 percent, *P* < .0001). Hospitalization data were available for all but six patients (0.03 percent) for whom social security numbers and statewide claims data were unavailable.

**TABLE 1:** Baseline characteristics of participants by trial

Characteristic	Trial 1 (n = 440) No. (%) or Mean ± SD	Trial 2 (n = 302) No. (%) or Mean ± SD	Trial 3 (n = 592) No. (%) or Mean ± SD	*P*‐value
Age	44.6 ± 12.3	56.4 ± 13.1	52.6 ± 11.1	<.0001
Female	264 (60.0%)	228 (75.5%)	370 (62.5%)	<.0001
African American	411 (93.4%)	286 (94.7%)	558 (94.3%)	.7414
Hispanic	10 (2.3%)	8 (2.7%)	11 (1.9%)	.7571
Employed	69 (15.7%)	42 (14.0%)	95 (16.1%)	.7071
Uninsured	305 (69.3%)	54 (17.9%)	13 (2.2%)	<.0001
Household income <$15 000	331 (81.7%)	135 (55.6%)	383 (73.5%)	<.0001
Low social support	57 (13.0%)	59 (19.6%)	133 (22.5%)	.0004
Alcohol overuse	113 (26.5%)	64 (21.4%)^b^	154 (29.0%)	.0579
Drug use	94 (21.6%)	34 (11.3%)	132 (22.5%)	.0002
Health literacy score^a^	1.9 ± 1.3	2.2 ± 1.3^c^	2.0 ± 1.2	.0232
Mean patient activation measure	59.4 ± 15.2	60.9 ± 13.5	60.1 ± 14.2	.3576
Self‐rated physical health	33.3 ± 10.7	35.7 ± 11.4^b^	33.8 ± 10.3	.0150
Self‐rated mental health	42.2 ± 12.6	44.8 ± 13.2^b^	42.7 ± 13.5	.0256
Delayed health need	225 (51.5%)	115 (38.5%)^c^	272 (46.5%)^c^	.0023
Unmet health need	172 (39.4%)	46 (15.4%)^c^	157 (26.8%)^c^	<.0001
One or more hospitalizations in previous 12 mo	396 (90.4%)	105 (35.0%)^c^	211 (35.8%)^c^	<.0001

Note

Scales from 1 to 100 unless otherwise indicated. For all variables, there was <5% missing data.

Abbreviations: BMI, body mass index; CHW, community health worker.

^a^ Measured on a scale of 5 (low) to 1 (high).

^b^ One patient value missing (ie, outcome and trial‐specific denominator = n−1).

^c^ Two patient values missing (ie, outcome and trial‐specific denominator = n−2).

### 3.2 Hospitalization outcomes

Hospitalization outcomes are summarized in Table 2. Over 9398 observed patient months, there was a significant reduction in total hospital days and hospital days per patient (849 days for 674 intervention patients vs 1258 days for 660 control patients, equating to 1.26 vs 1.90 bed days per patient; incidence rate ratio (IRR) 0.66; 95% CI 0.56‐0.77; *P* < .0001). This reduction was driven by a lower mean number of hospitalizations per patient (0.27 vs 0.34 in the intervention group vs control, IRR 0.80; 95% CI 0.72‐0.88; *P* < .0001), and among those hospitalized, a lower rate of multiple hospitalizations (5.0 percent vs 8.2 percent, OR 0.59; 95% CI 0.47‐0.75; *P* < .0001) and a shorter average length of stay (4.72 vs 5.57 days, IRR 0.83; 95% CI 0.71‐0.98; *P* = .0300) in the intervention group relative to the control group. Thirty‐day readmission rates were similar in the intervention and control groups (15.3 percent vs 17.6 percent, OR 0.85; 95% CI 0.56‐1.30; *P* = .45). Acuity of hospitalizations was also similar across groups, with an average DRG weight of 1.27 in both groups (*P* = .996). The group by trial interaction term was not statistically significant (*P* = .9316), suggesting homogenous effects across all three included trials.

**TABLE 2:** Hospital days, hospitalizations, and proportion of patients with multiple hospitalizations and hospitalizations outside the primary health system: regression analysis

Measure	Usual care (n = 660)	CHW intervention (n = 674)	Magnitude of difference	*P*‐value
Hospital days (total)	1258	849	‐	‐
Hospital days (per patient)	1.90	1.26	IRR 0.66 (95% CI 0.56‐0.77)	<.0001
# of Hospitalizations (total)	226	180	‐	‐
Mean # of Hospitalizations (per patient)	0.34	0.27	IRR 0.80 (95% CI 0.72‐0.88)	<.0001
Mean length of stay in days	5.57	4.72	IRR 0.83 (95% CI 0.71‐0.98)	.0300
% of Patients with multiple hospitalizations	8.2%	5.0%	OR 0.59 (95% CI 0.47‐0.75)	<.0001
% of Hospitalizations outside primary health system	34.8% (n = 178 hospitalizations)	18.8% (n = 138 hospitalizations)	OR 0.44 (95% CI 0.26‐0.73)	.0023
% of Patients with readmissions within 30 d	17.6%	15.3%	OR 0.85 (95% CI 0.56‐1.30)	.450
Mean diagnosis‐related group (DRG) weight of hospitalizations	1.27	1.27	Absolute difference 0.00 (95% CI −0.15‐0.15)	.9964

### 3.3 Fragmentation of hospital care

Only 18.8 percent of hospitalizations in the intervention group occurred outside the primary health system, compared to 34.8 percent in the control group (OR 0.44, 95% CI 0.26‐0.73, *P* = .0023), consistent with decreased fragmentation of care.

### 3.4 Timing of hospitalization

**FIGURE 2:**
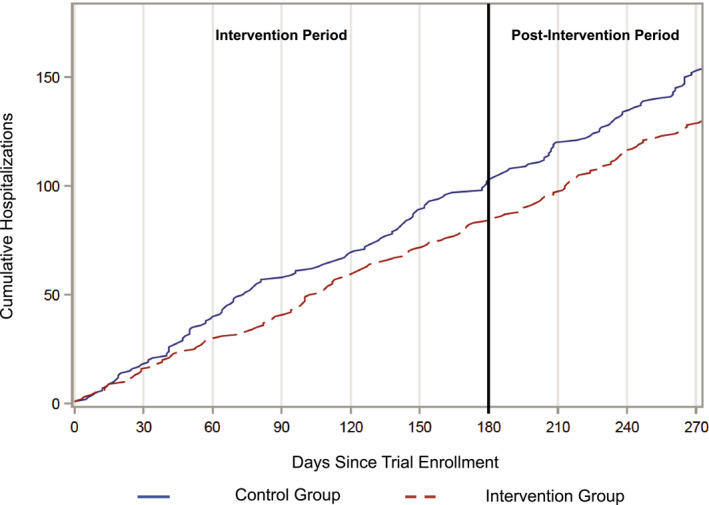
Cumulative Hospitalizations Over 9 months Following Enrollment [Color figure can be viewed at wileyonlinelibrary.com] *Notes: *This graph shows the cumulative number of hospitalizations for patients in the intervention control groups in the nine months following enrollment, including the six‐month IMPaCT intervention period and a three‐month postintervention period. This graph uses only data from trials 2 and 3, for which longitudinal hospitalization data were available.

Reductions in hospitalizations among patients in the intervention group relative to control were appreciable within the first months of the intervention period, with persistence through the end of the six‐month intervention and three months after cessation of the intervention (Figure 2).

## 4 DISCUSSION

This study pooled data from three randomized control trials spanning multiple clinical settings and showed that IMPaCT, a structured and standardized CHW intervention, reduced hospital utilization by multiple measures (bed days, hospitalizations, repeat hospitalizations, and length of stay), and also reduced fragmentation of hospital care. It comprises, what is to our knowledge, the largest analysis of randomized controlled data to demonstrate that a health system‐based social intervention can lead to significant and persistent decreases in acute care utilization. Our findings have several important implications for health systems and payers as they develop and implement interventions aimed at addressing patients’ social needs while simultaneously reducing healthcare costs.

First, this study adds to the growing evidence base suggesting that theory based, holistic approaches using tailored, longitudinal, relationship‐based support appear to be effective in reducing hospital utilization, a desirable but elusive goal for health system‐based social interventions.^12^, ^23^, ^24^, ^25^ This stands in contrast to other common approaches, including light‐touch social needs screening, augmented primary care, care management teams or domain‐specific interventions like transportation assistance.^9^, ^10^, ^11^, ^26^, ^27^, ^28^ Consistent and rigorous standardization of CHW hiring, training, work practices, caseloads, supervision, and infrastructure likely facilitated homogenous effects across trials. This intervention was able to reduce hospitalizations despite the fact that CHWs were explicitly trained and instructed not to keep patients out of the hospital, but rather to partner with patients and meet their needs, which were often social and recreational in nature. This suggests that patient‐centered interventions may paradoxically be more effective at reducing hospitalizations than interventions that are more narrowly focused on more conventional medical pathways such as improving adherence to medications or posthospital primary care appointments.^29^

Second, the study intervention reduced fragmentation of hospital care and appeared to create greater loyalty to the primary health system. Data from the original trials suggest that the IMPaCT intervention improved patients’ trust in their primary care teams, which likely strengthened patients’ perceived connection with the health system as a whole and may have encouraged them to choose these hospitals and clinics for their subsequent care.^13^, ^15^ This effect has important implications, as readmissions to different hospitals have been associated with a wide range of negative clinical and economic outcomes including longer lengths of stay, higher mortality rates, and increased hospitalization cost.^3^, ^4^, ^5^, ^6^

Third, our findings offer suggestions as to the mechanisms by which this CHW intervention may have reduced hospitalizations. It does not appear that CHWs reduced hospitalizations by delaying needed inpatient care, which would have led to a rebound in higher acuity hospitalizations after conclusion of the intervention, or by eliminating lower acuity hospitalizations, which would have led to increased acuity of hospitalizations after conclusion of the intervention. Rather, the persistent reduction in hospital utilization in the intervention arm suggests the intervention was successful in reducing hospitalizations primarily by addressing patients’ underlying socioeconomic and behavioral barriers to health and connecting them with long‐term medical care and social support. We hypothesize that patients in the intervention group may have had a decreased demand for acute inpatient services as a result of improvements in their chronic disease control, patient activation, and trust in their primary care teams, as well as decreased fragmentation of acute care.^13^, ^14^, ^15^ Future studies of health system‐based social interventions should assess the relative contributions of improvements in patient activation, chronic disease control, and patients’ perception of primary care to observed improvements in healthcare utilization.

This study has a number of limitations. Over 40 percent of eligible individuals declined participation, which may limit the external validity of our results. Hospitalization data were limited to acute care episodes within the state of Pennsylvania and did not include psychiatric admissions, emergency department visits, or hospitalizations outside of the state. Further, included trials had different lengths of follow‐up, although as they were all randomized controlled trials, there was no differential follow‐up by arm. The duration of follow‐up was limited, and further studies are needed to determine whether effects persist over longer time frames. While the included trials were conducted in a variety of different clinical settings and included patients with a range of sociobehavioral and hospitalization risk factors, all three were conducted in Philadelphia and in institutions with ties to the University of Pennsylvania Health System, the home to the IMPaCT program. In addition, all three enrolled participants who were disadvantaged and were predominantly African‐American. This could limit the generalizability of our findings, and future studies are needed to examine the effectiveness of this intervention in other geographic areas and with other patient populations. This study also has strengths, including the pooling of results from three distinct randomized clinical trials of a standardized intervention.

Overall, this study supports the benefit of a structured, standardized CHW intervention in reducing hospitalization and fragmentation of hospitalized care. These effects may have been achieved by letting patients “drive” the intervention in ways that produced lasting improvements in well‐being. These findings have particular implications for health systems and payers in search of evidence‐based methods to improve health outcomes by addressing social determinants of health.

## Supporting information

Supplementary Material

Tables S1‐S2
