# Supplementary material for: Effects of a standardized community health worker intervention on hospitalization among disadvantaged patients with multiple chronic conditions: A pooled analysis of three clinical trials
Source: Health Serv Res. 2020 Jul 8;55(Suppl 2):894–901. doi: 10.1111/1475-6773.13321 (PMC7518822; doi:10.1111/1475-6773.13321)
Supplement: Supplementary file 2 — Tables S1‐S2 [file HESR-55-894-s002.docx]

**Appendix**

**Table A1. Baseline Characteristics of Participants by Intervention Arm**

| **Characteristic** | **Usual Care (n = 660) No. (%) or Mean + SD** | **CHW Intervention (n = 674),  No. (%) or Mean + SD** |
| --- | --- | --- |
| Age | 50.3 **+** 12.8 | 51.3 + 12.8 |
| Female | 414 (62.7%) | 448 (66.5%) |
| African American | 623 (94.4%) | 632 (93.8%) |
| Hispanic | 11 (1.7%) | 18 (2.7%) |
| Employed | 92 (14.0%) | 114 (17.0%) |
| Uninsured | 175 (26.5%) | 197 (29.2%) |
| Household income  < $ 15 000 | 422 (72.9%) | 427 (72.4%) |
| Low social support | 118 (17.9%) | 131 (19.5%) |
| Alcohol overuse | 169 (27.1%) | 162 (25.4%) |
| Drug use | 127 (19.5%) | 133 (19.9%) |
| Health literacy score^a^ | 2.0 **+** 1.2 | 2.0 **+** 1.3 |
| Mean patient activation measure | 60.5 **+** 14.3 | 59.6 **+** 14.4 |
| Self-rated physical health | 33.9 **+** 10.6 | 34.2 **+** 10.9 |
| Self-rated mental health | 43.2 **+** 12.9 | 42.8 **+** 13.4 |
| Delayed health need | 295 (45.1%) | 317 (47.5%) |
| Unmet health need | 190 (29.1%) | 185 (27.7%) |
| One or more hospitalizations in previous 12 months | 354 (54.0%) | 358 (53.4%) |

Note: BMI = body mass index; CHW = community health worker. Scales from 1 to 100 unless otherwise indicated. For all variables, there was < 5% missing data. There were no significant differences between intervention and control group participant characteristics at $\alpha$=0.05.
a. Measured on a scale of 5 (low) to 1 (high)

**Table A2. Calculation of costs for Community Health Worker (CHW) Intervention**

| **COSTS PER CHW TEAM PER YEAR** |  |
| --- | --- |
| **Personnel** |  |
| Six Community Health Workers | $ 307,550 |
| Supervision and Support (Director, Manager and Coordinator) | $ 146,667 |
| TOTAL | $ 454,217 |
|  |  |
| **Equipment/Services** |  |
| Smartphones, Aircard Service | $ 6,739 |
| Laptops | $ 13,805 |
| Ongoing Training | $ 3,530 |
| Weekly Team Meetings | $ 1,434 |
| Patient Expenses | $ 2,500 |
| CHW Transportation | $ 6,732 |
| YMCA Memberships | $ 951 |
| Office Supplies | $ 1,821 |
| TOTAL | $ 37,512 |
|  |  |
| **Rent** |  |
| Office Space Rental | $ 14,070 |
|  |  |
| TOTAL: Direct Costs per CHW Team per Year | $ 505,800 |
| Indirect Rate | 0.12 |
| TOTAL: Yearly Costs per CHW Team per Year | $ 566,496 |
|  |  |
| **INTERVENTION COSTS** |  |
| # Patients in Intervention Arm | 674 |
| # Patients managed per CHW Team per Year | 378 |
| # CHW Team-Years required for Intervention | 1.78 |
|  |  |
| **TOTAL: Intervention Cost** | $ 1,010,155 |
| **TOTAL: Intervention Cost Per Patient** | $ 1,499 |
| *Source: Author’s analysis* | |
